# Axial elongation measured by long scan depth optical coherence tomography during pilocarpine-induced accommodation in intraocular lens-implanted eyes

**DOI:** 10.1038/s41598-018-19910-0

**Published:** 2018-01-31

**Authors:** Yilei Shao, Qiuruo Jiang, Di Hu, Lingmin Zhang, Meixiao Shen, Shenghai Huang, Lin Leng, Yimin Yuan, Qi Chen, Dexi Zhu, Jianhua Wang, Fan Lu

**Affiliations:** 1 School of Ophthalmology and Optometry, Wenzhou Medical University, Wenzhou, Zhejiang China; 2 Bascom Palmer Eye Institute, Department of Ophthalmology, Miller School of Medicine, University of Miami, Miami, FL USA

## Abstract

We used an ultra-long scan depth optical coherence tomography (UL-OCT) system to investigate changes in axial biometry of pseudophakic eyes during pilocarpine- induced accommodation. The right eyes from 25 healthy subjects (age range 49 to 84 years) with an intraocular lens (IOL) were imaged twice in the non-accommodative and the accommodative states. A custom-built UL-OCT instrument imaged the whole eye. Then accommodation was induced by two drops of 0.5% pilocarpine hydrochloride separated by a 5-minute interval. Following the same protocol, images were acquired again 30 minutes after the first drop. The central corneal thickness (CCT), anterior chamber depth (ACD), IOL thickness (IOLT), and vitreous length (VL) were obtained using custom automated software. The axial length (AL) was calculated by summing the CCT, ACD, IOLT, and VL. With accommodation, ACD increased by +0.08 ± 0.09 mm, while the VL decreased by −0.04 ± 0.09 mm (paired t-test each, P<0.05). CCT and IOLT remained constant during accommodation (P > 0.05). The non-accommodative AL was 23.47 ± 0.93 mm, and it increased by +0.04 ± 0.04 mm after accommodation (P<0.01). The AL increased and the IOL moved backward during pilocarpine-induced accommodation in pseudophakic eyes.

## Introduction

Accommodation in the human eye is a dynamic process that originates from the contraction of ciliary muscle and results in the reshaping of the crystalline lens. According to the classic theory by Helmholtz^1^, the accommodation occurs as the crystalline lens thickens and moves forward toward the anterior surface. In this process, the cornea changes only slightly^2^. However, whether or not the posterior segment of the eye, including the vitreous chamber and the retina, changes during accommodation remains controversial.

Changes of ocular axial length (AL) with accommodation are still a matter of controversy. Some studies found a small but significant transient increase in AL accompanying accommodation in youth and elderly people^3–12^, but others reported the AL remained constant during accommodation^13,14^. Several techniques have been used to measure ocular AL during accommodation. With A-scan ultrasound, which requires contact of the probe on the corneal surface, the AL was reported to increase^3^ or remain constant^13,14^ during accommodation. In these studies, the effect of placing the ultrasound probe on the cornea could have influenced the results. The IOLMaster, which uses an average ocular refractive index that converts optical distances into geometric distances, has also been used to evaluate axial elongation during accommodation^5,7,8^. As an improvement, the Lenstar measures each compartmental distance^6,9,10,12^. Similarly, we^11,15,16^ and others^17^ previously developed ultra-long scan depth optical coherence tomography (UL-OCT) instruments that can measure the dimensions of each component of the whole eye individually during accommodation.

However, changes in the shape of the crystalline lens during accommodation may result in changes of the effective refractive index. In turn, this could lead to a change of optical path distance and subsequent deviation of the measurement from the true change in AL^18–21^. That was the main limitation of the previous studies using IOLMaster and Lenstar^4–10,12^. Some research reported that the refractive index increases by +0.001 to +0.0015 per diopter (D) during accommodation^20,22–24^, while other studies found no change in the lens gradient refractive index^18,19^. Recent studies have attempted to overcome this potential error by using the ocular biometry data of individual subjects to calculate the error in measured AL^6,9,10^ or measuring AL immediately following accommodation or after accommodation ceased^8^. Because the exact change in refractive index of the crystalline lens during accommodation is not known, the values of AL during accommodation in those studies still may not be the true value at that time.

This cross-sectional and population-based study was performed using UL-OCT to investigate changes in axial biometry of pseudophakic eyes during pilocarpine-induced accommodation. Changes in axial ocular biometry, including the central corneal thickness (CCT), anterior chamber depth (ACD), intraocular lens thickness (IOLT), and vitreous length (VL) were analyzed. To avoid the measurement artifacts potentially caused by changes in the refractive index of the natural crystalline lens^20,22–24^, we enrolled patients with implanted intraocular lenses (IOLs), which have a constant refractive index. Considering that the ciliary muscle in aging human eyes still has the capability of contraction^25–27^, we used UL–OCT to evaluate the change in ocular AL during pilocarpine-induced accommodation in patients with implanted IOLs.

## Methods

### Study population and Ethics Statement

The study was approved by the Wenzhou Medical University review board, and written informed consent was provided by each subject. All patients were treated in accordance with the tenets of the Declaration of Helsinki. The right eyes of 25 patients (9 males and 16 females) were included in the study. Their ages ranged from 49 to 84 years (mean ± standard deviation: 68.3 ± 8.4 years). All subjects were recruited at the Affiliated Eye Hospital of Wenzhou Medical University, Wenzhou, Zhejiang, China. Cataracts were diagnosed in both eyes for each subject, and at least one eye underwent phacoemulsification surgery with implantation of an IOL (Akreos MI 60 aspheric micro-incision intraocular lens, Bausch & Lomb, Rochester, NY, USA). The UL-OCT measurements were performed 1 to 3 months after the operation. Exclusion criteria included post-operative best corrected visual acuity less than 0.5, inflammation, previous ocular surgery except cataract extraction and IOL implantation, contact lens wear, or other current ocular or systemic diseases.

### Instruments

As reported previously^11,15,16^, we used a custom-built UL-OCT instrument to image the whole eye from cornea to retina through the pupil. Briefly, the light source had a center wavelength of 840 nm with a bandwidth of 50 nm. The spectrometer included a collimating lens with a focal length of 50 mm, an 1,800 line/mm transmission grating, an enlargement lens with a focal length of 240 mm, and a line array complementary metal-oxide-semiconductor camera (Basler Sprint spL4096-140k; Basler AG, Ahrensburg, Germany). The scan depth was 11.964 mm, while the axial resolution was 7 µm in air. To extend the effective scan depth, a switchable reference arm with 3 mirrors at different distances was utilized to sequentially acquire 3 frames. The two frames from the first and second reference mirrors were overlapped to obtain the entire anterior segment through the pupil, while the third mirror was for imaging the retina. The optical path distance between the first and the third mirror was 33.160 mm in air. Thus the whole eye image could be obtained by reconstruction of the 3 frames.

A vision target consisting of a white Snellen letter E with a logMAR visual acuity size of 0.4 was displayed by a light-emitting diode on a black background. The target was mounted onto the sample arm and was congruent to the beam from the UL-OCT light source by a hot mirror. During image acquisition, the patients were asked to fix on the target with the measured eye while the other eye was covered. A cross-hair live view at both the horizontal and vertical meridians was applied to monitor that the measured eye was positioned correctly. This approach ensured that all scans were at the same sagittal section and that each measurement was conducted on the visual axis of the eye.

### Study Design and Examination

The scan speed of UL-OCT was set as 10,000 A-lines/s and each frame consisted of 1,024 A-lines. Fifteen frames were obtained at baseline in approximate 1.5 seconds for each subject. The subjects were asked to take a short rest (approximate 30 s) before the measurement was repeated. Then accommodation was induced by 2 drops of 0.5% pilocarpine hydrochloride separated by a 5-minute interval. Thirty minutes after the first drop, the images were acquired again with the same protocol. All measurements were performed by the same researcher (DH).

### Data Analysis

Automated custom software was used to segment the full-range ocular images as described in our previous papers^11,16^. At first, every 3 frames were reconstructed to obtain one whole eye image. After removing the saturated A-lines resulting from the specular reflection on the corneal apex, longitudinal reflectivity profiles were obtained from the 20 central A-lines of each image. The structural boundary of each compartment was detected from the corneal anterior surface to the retinal pigment epithelium. Optical path lengths through the central cornea, anterior chamber, IOL, and vitreous were calculated. The geometric length of each compartment was determined by dividing the optical path length by the refractive index of each structure (refractive indices of the cornea, aqueous humor, IOL and vitreous were 1.387^28^, 1.342^29^, 1.460^30^, and 1.341^29^ at 840 nm wavelength, respectively). The AL of the whole eye along the visual axis was determined as the sum of CCT, ACD, IOLT, and VL. The results from five images were averaged as a single measurement for further analysis.

Descriptive statistics were determined as means ± standard deviations. Bland-Altman plots were used to determine interclass correlation coefficients (ICCs) as a measure of agreement between the repeated measurements of each compartment. Moreover, the coefficients of repeatability (CoRs) were determined by dividing the two standard deviations of the differences between the repeated measurements by the average of the two repeated measurements^31^. After determining that the data for each variable was normally distributed, two-tailed paired t-tests were used for statistical comparisons of lengths between the baseline and accommodative states, and P < 0.05 was considered statistically significant.

### Validation for the measurement of the axial elongation using UL-OCT

**Figure 1:**
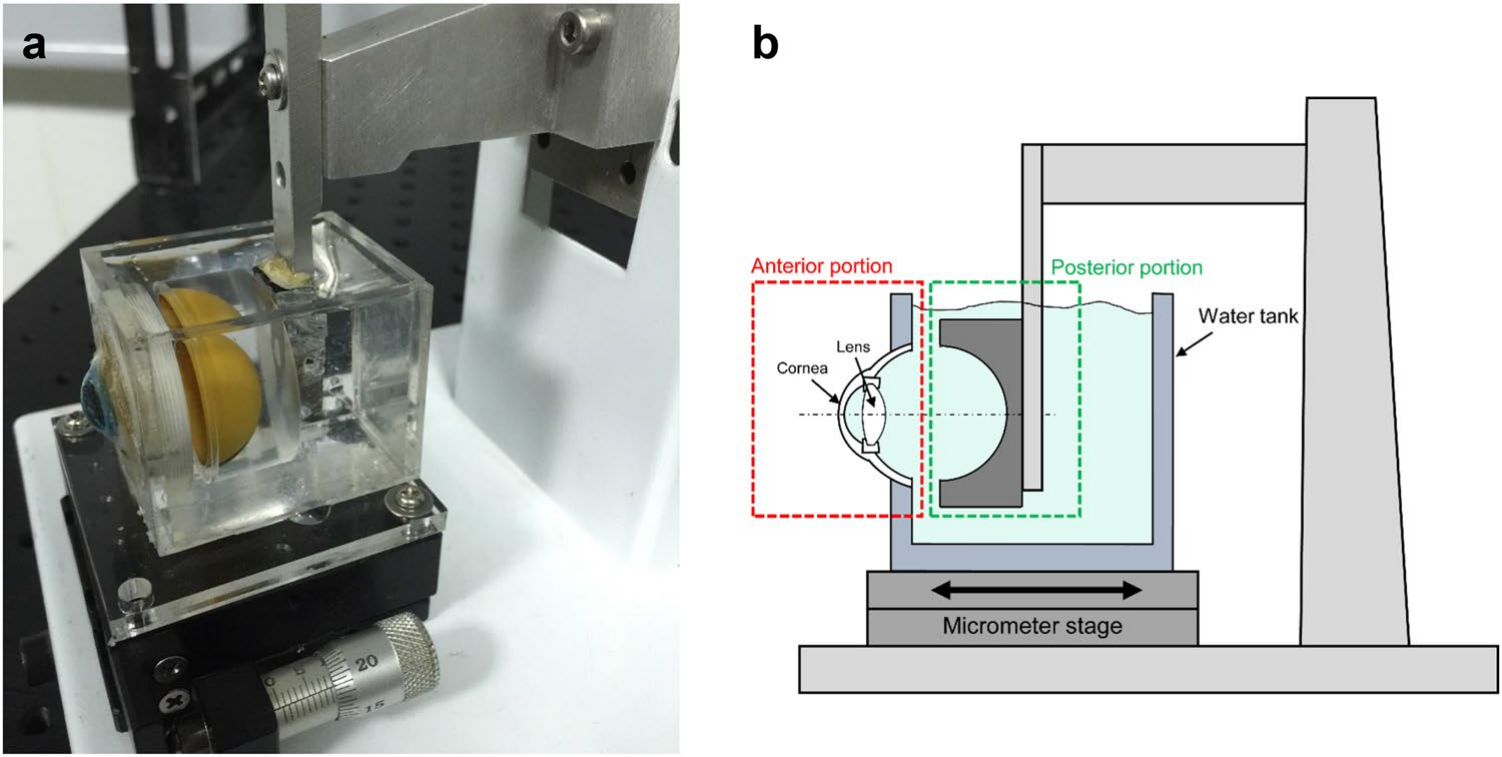
Photograph (**a**) and schematic diagram (**b**) of the physical model eye. A PMMA shell and lens simulated the cornea and crystalline lens. The anterior portion (cornea and lens) was inserted into a water tank, which was mounted on a micrometer stage that was moved along the ocular axis. The posterior portion was fixed on the platform by a holder.

To validate the measurements of the axial elongation using UL-OCT, we used a modified physical model eye (OEMI-7; Ocular Instruments, Inc., Bellevue, WA, USA). It consisted of a poly-methyl methacrylate (PMMA) water-cell model that simulated the cornea and the lens (Fig. 1). The model eye was separated into two portions. The anterior portion, including the cornea and lens, was mounted within a water tank that was secured to a micrometer stage. To elongate the model eye, the stage was advanced along the ocular axis, extending the anterior portion while the posterior portion remained fixed in position. The model axis was elongated from 0 to 200 µm in steps of 25 µm, and UL-OCT images along the optical axis were captured after each step. To calculate changes in compartment thickness of the model eye, we used 1.485 as the refractive index of PMMA and 1.326 for water at a wavelength of 840 nm.

### Accuracy of the axial elongation measurements by UL-OCT

**Figure 2:**
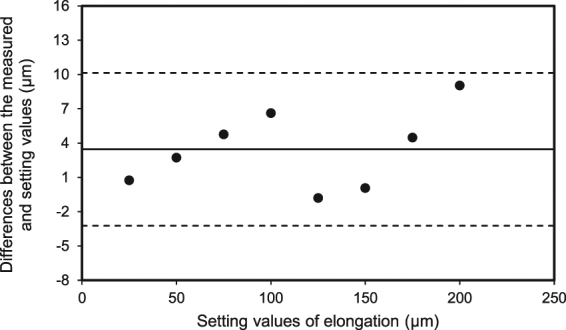
Bland-Altman plots of the differences between the UL-OCT-measured and pre-set elongation provided by the micrometer stage. Values on the vertical axis correspond to the differences between the measurements and setting values, and values on the horizontal axis correspond to the setting values. Solid and dashed lines indicate the mean difference and 95% confidence intervals, respectively.

Using the eye model, we measured the differences between the pre-set elongation values and the UL-OCT-determined values. The mean difference was 4 ± 3 μm (range, −1 to 9 μm), and a Bland-Altman plot (Fig. 2) showed that the differences were within the 95% confidential interval.

## Results

### Changes in ocular axial biometry during pilocarpine-induced accommodation

**Figure 3:**
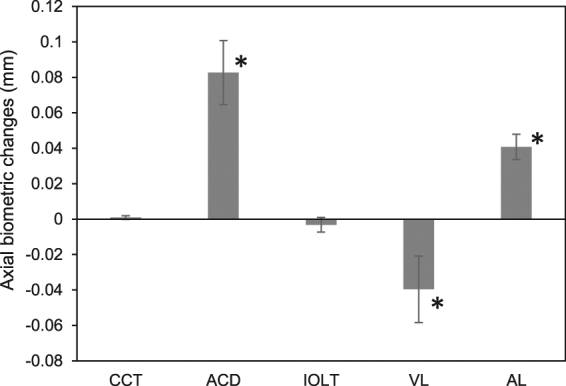
Changes in axial biometry of pseudophakic eyes during pilocarpine-induced accommodation. CCT, central corneal thickness; ACD, anterior chamber depth; IOLT, intraocular lens thickness; VL, vitreous length; AL, axial length; *P < 0.05, comparisons between resting and accommodative states by two-tailed paired t-tests. Bar = 1 standard error.

After pilocarpine-induced accommodation, the ACD increased from the baseline of 4.24 ± 0.31 mm to 4.33 ± 0.31 mm, an increase of +0.08 ± 0.09 mm (Table 1, Fig. 3, P < 0.01, paired t-test). The VL decreased from 17.85 ± 0.94 mm to 17.81 ± 0.95 mm, a decrease of −0.04 ± 0.09 (Table 1, Fig. 3, P < 0.05, paired t-test). CCT and IOLT remained constant during accommodation (P > 0.05). Finally, the AL increased from 23.47 ± 0.93 mm at baseline to 23.51 ± 0.94 mm after accommodation. The overall increase was + 0.04 ± 0.04 mm (Table 1, Fig. 3, P < 0.01, paired t-test).

**Table 1:** Axial biometry of pseudophakic eyes as measured by ultra-long scan depth OCT.

		CCT^*^	ACD^*^	IOLT^*^	VL^*^	AL^*^
Resting	V1	0.55 ± 0.03	4.25 ± 0.31	0.82 ± 0.03	17.85 ± 0.93	23.47 ± 0.93
	V2	0.55 ± 0.03	4.24 ± 0.31	0.83 ± 0.03	17.85 ± 0.94	23.47 ± 0.93
	mean	0.55 ± 0.03	4.24 ± 0.31	0.82 ± 0.03	17.85 ± 0.94	23.47 ± 0.93
	Dif	0.00 ± 0.01	0.01 ± 0.04	−0.01 ± 0.01	−0.00 ± 0.04	0.00 ± 0.02
	CoR	2.11%	1.71%	3.54%	0.46%	0.16%
	ICC	0.991	0.997	0.928	1.000	1.000
Accommodation	V1	0.55 ± 0.03	4.33 ± 0.31	0.82 ± 0.02	17.81 ± 0.95	23.51 ± 0.93
	V2	0.55 ± 0.03	4.33 ± 0.32	0.82 ± 0.02	17.81 ± 0.95	23.51 ± 0.94
	mean	0.55 ± 0.03	4.33 ± 0.31	0.82 ± 0.02	17.81 ± 0.95	23.51 ± 0.94
	Dif	−0.00 ± 0.01	−0.00 ± 0.04	−0.00 ± 0.02	0.00 ± 0.03	−0.00 ± 0.03
	CoR	2.81%	1.88%	4.22%	0.36%	0.23%
	ICC	0.985	0.996	0.841	1.000	1.000
DIF		+0.00 ± 0.01	+0.08 ± 0.09	−0.00 ± 0.02	−0.04 ± 0.09	+0.04 ± 0.04
P value**		0.39	**<0.01**	0.44	**<0.05**	**<0.01**

^*^All measurements were in millimeters; CCT, central corneal thickness; ACD, anterior chamber depth; IOLT, thickness of the intraocular lens; VL, vitreous length; AL, axial length.

**Comparisons between resting and accommodative states by two-tailed paired t-tests.

V1 and V2: 1st and 2nd measurements; Dif: difference between the V1 and V2; CoR: coefficient of repeatability; ICC: interclass correlation coefficients; DIF: Difference in values between the resting and accommodative states.

### Repeatability of ocular axial biometry measurements by UL-OCT

**Figure 4:**
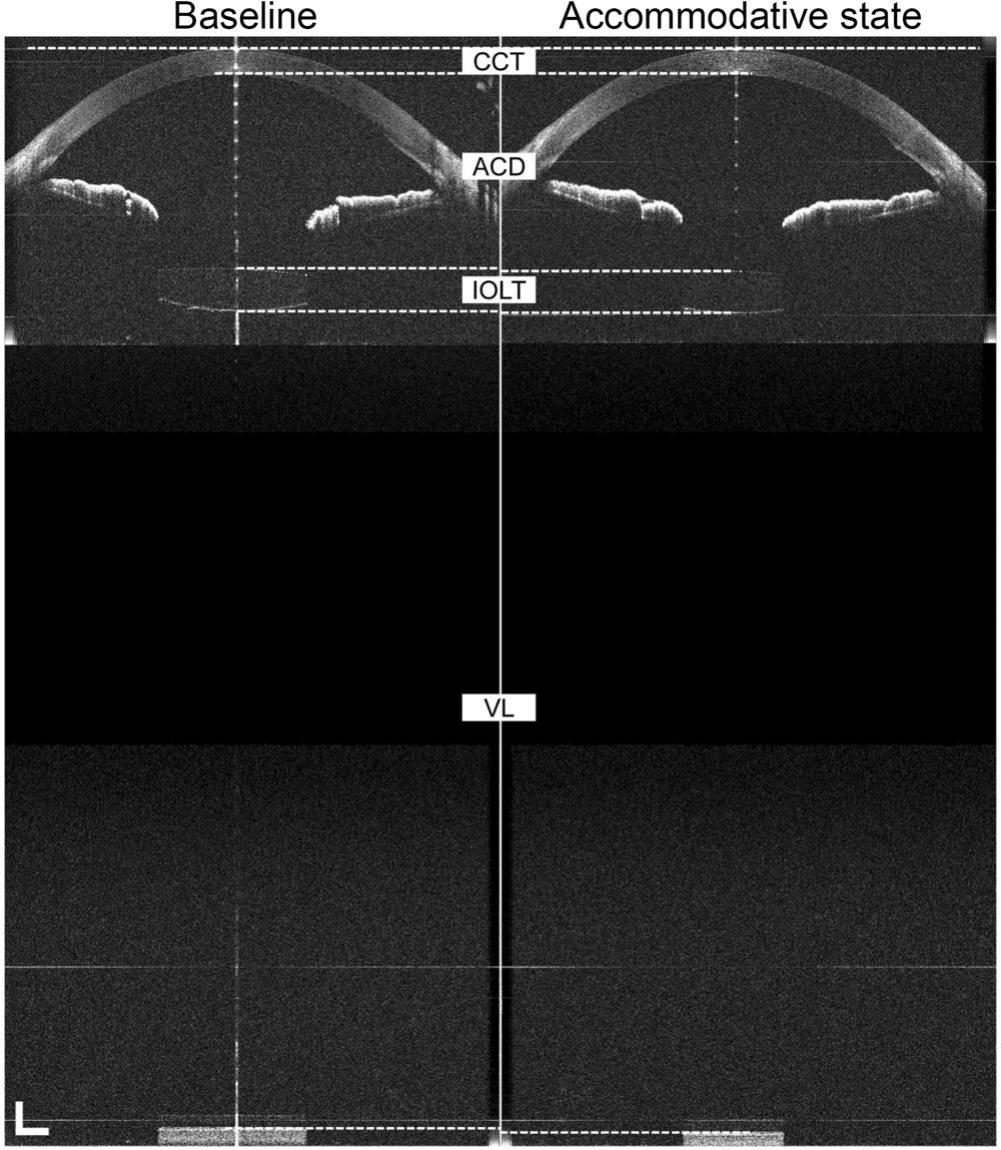
Whole eye from the cornea to the retina through pupil of a 49-year-old subject before and after pilocarpine-induced accommodation. The images were acquired by ultra-long scan depth OCT. The left side was obtained at the resting state, while the right side was taken during accommodation. In this subject, the IOL exhibited a backward movement of 0.086 mm, while the axial length increased from 24.034 to 24.069 mm. CCT, central corneal thickness; ACD, anterior chamber depth; IOLT, intraocular lens thickness; VL, vitreous length; AL, axial length. Bar = 1 mm.

**Figure 5:**
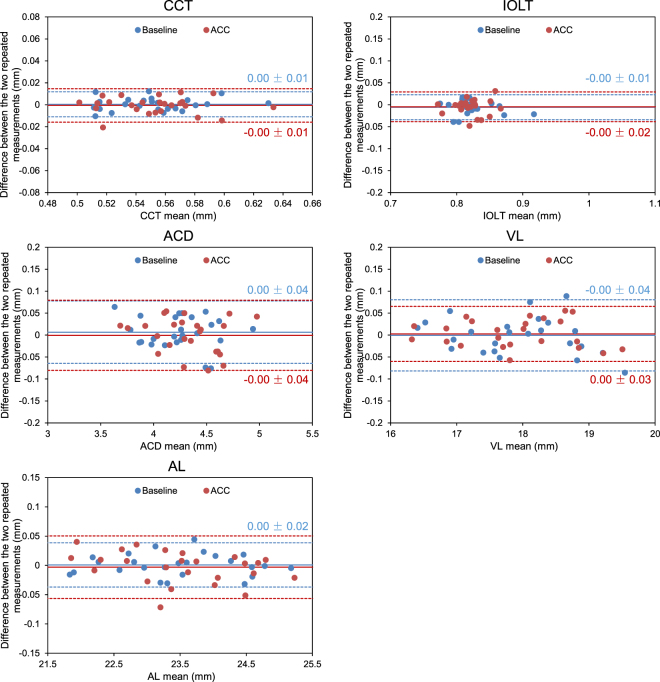
Bland-Altman plots of the difference between the two repeated measurements of whole eye axial biometry in 25 eyes before (blue, Baseline) and after (red, ACC) pilocarpine-induced accommodation. Values on the vertical axis correspond to the difference between the two measurements, and values on the horizontal axis correspond to the average of the two measurements. Solid and dashed lines indicate the mean differences and 95% confidence intervals, respectively. Blue and red values were the mean difference ± standard deviation of the repeated measurements at the baseline and the accommodative states, respectively. CCT, central corneal thickness; ACD, anterior chamber depth; IOLT, intraocular lens thickness; VL, vitreous length; AL, axial length.

Images of the whole eye from the cornea, through the pupil, to the retina were successfully obtained by UL-OCT at both baseline and accommodative states (Fig. 4). Bland-Altman plots of the differences between the repeated measurements for each parameter were constructed (Fig. 5). At baseline in the resting state, the CoRs of the parameters ranged from 0.16% to 3.54%, and the ICCs ranged from 0.928 to 1.000 (Table 1). After accommodation was induced by pilocarpine, the CoRs ranged from 0.23% to 4.22%, and the ICCs ranged from 0.841 to 1.000 (Table 1).

## Discussion

In this study, we used UL-OCT to demonstrate that the ocular axial biometry undergoes significant change during pilocarpine-induced accommodation in pseudophakic eyes. Sheppard & Davies^12,26^ reported the morphologic changes in the aging ciliary muscle (aged from 19–70 years), but those changes appeared not to affect the contractile ability of the muscle during accommodation. Strenk *et al*.^32^ also found that the accommodation-related change in ciliary muscle ring was undiminished by age (22–91 years) or IOL implantation. Therefore, the ciliary muscle seems to retain the ability for contraction throughout life, even though the accommodative amplitude decreases with age. The work of Park *et al*.^33^ showed that the centripetal movement of human ciliary body increases significantly after cataract extraction. Thus, even though a recent study by Croft *et al*.^34^ suggested the accommodative ciliary muscle contraction declined with age in phakic eyes, the results might be different in pseudophakic eyes.

Pilocarpine causes contraction of the ciliary muscle, and Koeppl *et al*.^35^ demonstrated that it acts as a “superstimulus” in presbyopic phakic eyes, resulting in an “unphysiological” response. The authors hypothesized that pilocarpine stimulation may produce a “physiological” accommodation in both young and presbyopic subjects. However the stimulus-driven accommodation is sub-maximal in presbyopes and may be insufficient to cause detectable axial elongation. Therefore, we used pilocarpine, even though it could act “unphysiologically” but elicit a superstimulus effect, to ensure adequate contraction of the ciliary muscle.

The high accuracy of the UL-OCT instrument was validated by measuring the axial elongation of a physical eye model that was stretched by mechanical tension. The mean absolute discrepancy between the UL-OCT length measurements and the micrometer setting values was 4 µm. With the UL-OCT instrument used in our study, the CoRs and ICCs for axial biometry of the pseudophakic eyes are consistent with the excellent repeatability of this instrument that we previously reported^11,16^. This high level of accuracy and repeatability assures that the eye elongation was associated with the pilocarpine-induced accommodation rather than any measurement error. The ICCs and CORs of the IOLT were relatively worse than for other parameters. The explanation may be associated with the probable occurrence of IOL tilt that could cause the measurements to diverge slightly from the central axis of the IOL.

Previous studies have investigated the change in AL with accommodation (Table 2)^3–6,9–12^. For instance, Woodman *et al*.^9^ found a mean increase in the AL of +0.020 mm in phakic eyes for a 4.0 D accommodation. Mallen *et al*.^5^ reported a +0.014 mm increase with 2.0 D of accommodation, and +0.037 mm increase with 6.0 D of accomodation. Using a similar UL-OCT system in our previous study^11^, we demonstrated axial elongation of +0.026 mm during 6.0 D of accommodation. Most of the previous studies used the near target as the accommodative stimulus^3,5,6,9–12^. Here, we induced accommodation by pharmacological stimulation of the ciliary muscle in pseudophakic eyes of older subjects (49–84 years of age) and measured an increase in AL of +0.04 mm. Lister *et al*.^36^ found that the pilocarpine instillation significant increased lens thickness for both young and older groups, indicating that pharmacological stimulation influenced both young and older subjects. The exact level of accommodation due to the pharmacologically induced ciliary muscle contraction stimulated was unknown, and the biometrics of the older and pseudophakic eyes might not be similar to the younger phakic eyes, as reported in Lister’s study^36^. Though not directly comparable, this value was slightly larger than that reported by the other studies.

**Table 2:** Summary of previous studies investigating the changes in axial length during accommodation.

Study	Technique	Sample size	Age	Accommodation	Increase in axial length^48^	
Current study	UL-OCT	25	49–84	Pilocarpine induced	0.04 ± 0.04	
Shum *et al*.^3^	Ultrasonic biometer	106	18–22	3.0 D	0.06 ± 0.01	
Drexler *et al*.^4^	Custom PCI	EM: 11 M: 12	21–30	Near point	EM: 0.013 ± 0.003	M: 0.005 ± 0.002
Mallen *et al*.^5^	IOLMaster	EM: 30 M: 30	EM: 21.4 ± 2.0 M: 21.5 ± 2.1	2.0 D 4.0 D 6.0 D	EM: 0.014 ± 0.019	M: 0.019 ± 0.020
					EM: 0.026 ± 0.021	M: 0.037 ± 0.026
					EM: 0.037 ± 0.027	M: 0.058 ± 0.037
Read *et al*.^6^	Lenstar	EM: 19 M: 21	18–33	3.0 D 6.0 D	EM: 0.013 ± 0.013	M: 0.011 ± 0.012
					EM: 0.025 ± 0.015	M: 0.023 ± 0.023
Woodman *et al*.^9^	Lenstar	EM: 22 M: 37	18–30	4.0 D	EM: 0.006 ± 0.022	M: 0.022 ± 0.034
Ghosh *et al*.^10^	Lenstar	EM: 10 M: 10	18–30	2.5 D	0.007 ± 0.008	
Laughten *et al*.^12^	Lenstar	EM: 10 M: 10	34–41	4.5 D	EM: 0.002 ± 0.006*	M: 0.005 ± 0.004*
Zhong *et al*.^11^	UL-OCT	21	19–35	6.0 D	0.026 ± 0.013	

PCI: Partial coherence interferometry; UL-OCT: Ultra-long scan depth optical coherence tomography; EM: Emmetropes; M: Myopes; D: diopters.

*Change per diopter of accommodation.

We enrolled IOL-implanted patients because the refractive index of each component of their eyes was constant. Thus the absence of refractive index changes in each component of the eye during accommodation ensured that it could not have influenced the measurement of axial elongation. In contrast, Mallen *et al*.^5^ used a commercial IOLMaster device for AL measurements that did not include individual optical correction for each compartment. Thus, the measured changes could have been overestimated due to the potential error associated with lens thickening during accommodation^21^.

The possible alteration of the crystalline lens refractive index may also cause another measurement error^20,22,24^. The effects of accommodation on the refractive index of the lens are contentious. Using a ray trace method, Gullstrand deduced that the reshaping of the crystalline lens during accommodation causes the fibers inside the lens to slide on each other and change the equivalent refractive index of the lens from 1.413 to 1.424^24^. Based on *in vivo* examination, Kasthurirangan *et al*.^20^ found a significant increase in the size of lens central plateau region during accommodation with constant refractive index. This resulted in an increase of the equvilant refractive index of the lens. However, Hermans *et al*.^18^ suggested the equivalent refractive index of the human lens might remain unchanged with accommodation, and Jones *et al*.^19^ found a slight, but not significant, decrease in central refractive index with accommodation. Those potential changes in the refractive index of the crystalline lens were not taken into account in previous studies^4–12^. Misestimating the refractive index during the accommodative state will result in a deviation of the measurement from the lens true thickness. This will then result in misestimatation of the AL and the axial elongation during accommodation. This measurement artifact was avoided in our subjects because the IOLs had a constant refractive index.

To explain the elongation of the eye during accommodation, some researchers proposed that the force of ciliary muscle contraction produces an inward pull on the choroid and sclera adjacent to the ciliary muscle^4,5^. The force of the contraction would decrease the circumference of the globe at the equator, forcing the rearward movement of the posterior portion of the globe to maintain a constant ocular volume. Coudrillier *et al*.^37^ found that the human posterior sclera stiffens with age, which could limit the scleral stretching under the stress. This suggests that the aging human eyes enrolled in the present study may respond differently to elongation forces than eyes in young people. The elongation of AL found in the current study was greater than previous studies that enrolled younger subjects. The pilocarpine we used could have acted as a superstimulus for ciliary muscle contraction, which may have caused a larger increase of AL than that during physiological accommodation. Another explanation for the anatomical change may be a thinning of the choroid during accommodation. Using the Lenstar, Woodman *et al*.^9^ observed a decrease in choroidal thickness that had a weak but significant negative correlation with the changes in AL, although the magnitude of thinning in choroid was only 38% of the increase in AL. Using OCT, Woodman *et al*.^38^ confirmed the decrease of choroidal thickness with accommodation. As with the sclera, the elasticity of the choroid also decreases with age^39,40^. However, compared to sclera, the choroid is a much more elastic tissue that is comprised of blood vessels, melanocytes, fibroblasts, resident immunocompetent cells, and supporting collagenous and elastic connective tissue. Thus the thinning of the choroid is unlikely to be solely responsible for eye elongation during accommodation; however, it may contribute to the stretching of the globe, especially in elderly subjects with stiffer scleras.

In addition to eye elongation, we found a significant increase in the ACD during the pilocarpine-induced accommodation. The increase in ACD results from movement of the IOL in the posterior direction, as previously reported by others^41,42^. However in phakic eyes, the crystalline lens moves forward during accommodation and therefore causes a decrease in the ACD^11,43–47^.

There were limitations in the current study. First, we used pilocarpine to induce contraction of the ciliary muscle in older subjects with pseudophakic eyes. Typically, the accommodative ability in phakic individuals of this age group is lower than that of young subjects. For this reason, we chose to induce accommodation pharmacologically to ensure a strong contraction of the ciliary muscle. While this mode of accommodation might not be precisely like natural accommodation during near work, the utilization of pilocarpine was justifid to achieve a strong ciliary muscle contraction in the older subjects. Second, the results may have been affected by the different times of measurements after date of surgery. A previous study^31^ showed that the reduction of ciliary ring diameter during accommodation due to the contraction of ciliary muscle was 0.11 mm at one month after phacoemulsification surgery. The changes in diameter slightly increased to 0.14 mm at two months and to 0.18 mm at 12 months after surgery. These changes were stable at all of the succeeding follow-up examinations, indicating that the capability of ciliary muscle was restored after surgery and maintained for at least for one year. For our study, period between phacoemulsification surgery and measurement of the ocular axial parameters by UL-OCT was 1–3 months. Thus, the influence of the time after surgery for our subjects was probably small. The third limitation arose because the telecentric optical design of the scanning probe could not obtain detailed images of the retina. Thus, the retina appeared as a straight line rather than having the normal curvature. For that reason, we asked the subjects to fixate on the target that was adjacent to the UL-OCT beam to ensure that the measuring beam was aimed at the visual axis.

In conclusion, we used UL-OCT to demonstrate that the ocular AL increased with pilocarpine-induced accommodation in pseudophakic eyes of older subjects. Because the IOL has a constant refractive index, it is clear that the axial elongation was due to accommodation rather than a measurement artifact associated with a change in the refractive index of the natural crystalline lens. In addition to the elongation of the AL during accommodation, there was a backward movement of the IOL. These findings provide a more precise understanding of changes in ocular biometric properties with accommodation. This new information may help to establish a link between near work and longer-term axial elongation of the eye.

## References

[1] H.von Helmholtz. Uber die akkommodation des auges. *Archiv Ophthalmol* **1**, 1–74 (1855).

[2] He JC, Gwiazda J, Thorn F, Held R, Huang W (2003). Change in corneal shape and corneal wave-front aberrations with accommodation. Journal of vision.

[3] Shum PJ, Ko LS, Ng CL, Lin SL (1993). A biometric study of ocular changes during accommodation. American journal of ophthalmology.

[4] Drexler W, Findl O, Schmetterer L, Hitzenberger CK, Fercher AF (1998). Eye elongation during accommodation in humans: differences between emmetropes and myopes. Investigative ophthalmology & visual science.

[5] Mallen EA, Kashyap P, Hampson KM (2006). Transient Axial Length Change during the Accommodation Response in Young Adults. Investigative ophthalmology & visual science.

[6] Read SA, Collins MJ, Woodman EC, Cheong SH (2010). Axial length changes during accommodation in myopes and emmetropes. Optometry and vision science: official publication of the American Academy of Optometry.

[7] Alderson A, Mankowska A, Cufflin MP, Mallen EA (2011). Simultaneous measurement of objective refraction, accommodation response and axial length of the human eye. Ophthalmic & physiological optics: the journal of the British College of Ophthalmic Opticians (Optometrists).

[8] Woodman EC (2011). Axial elongation following prolonged near work in myopes and emmetropes. The British journal of ophthalmology.

[9] Woodman EC, Read SA, Collins MJ (2012). Axial length and choroidal thickness changes accompanying prolonged accommodation in myopes and emmetropes. Vision research.

[10] Ghosh A, Collins MJ, Read SA, Davis BA, Chatterjee P (2014). Axial elongation associated with biomechanical factors during near work. Optometry and vision science: official publication of the American Academy of Optometry.

[11] Zhong J (2014). Whole eye axial biometry during accommodation using ultra-long scan depth optical coherence tomography. American journal of ophthalmology.

[12] Laughton DS, Sheppard AL, Davies LN (2016). A longitudinal study of accommodative changes in biometry during incipient presbyopia. Ophthalmic & physiological optics: the journal of the British College of Ophthalmic Opticians (Optometrists).

[13] van der Heijde GL, Weber J (1989). Accommodation used to determine ultrasound velocity in the human lens. Optometry and vision science: official publication of the American Academy of Optometry.

[14] Garner LF, Yap MK (1997). Changes in ocular dimensions and refraction with accommodation. Ophthalmic & physiological optics: the journal of the British College of Ophthalmic Opticians (Optometrists).

[15] Tao A (2013). Versatile optical coherence tomography for imaging the human eye. Biomedical optics express.

[16] Zhong J (2014). Axial biometry of the entire eye using ultra-long scan depth optical coherence tomography. American journal of ophthalmology.

[17] Ruggeri M (2012). Imaging and full-length biometry of the eye during accommodation using spectral domain OCT with an optical switch. Biomedical optics express.

[18] Hermans EA, Dubbelman M, Van der Heijde R, Heethaar RM (2008). Equivalent refractive index of the human lens upon accommodative response. Optometry and vision science: official publication of the American Academy of Optometry.

[19] Jones CE, Atchison DA, Pope JM (2007). Changes in lens dimensions and refractive index with age and accommodation. Optometry and vision science: official publication of the American Academy of Optometry.

[20] Kasthurirangan S, Markwell EL, Atchison DA, Pope JM (2008). *In vivo* study of changes in refractive index distribution in the human crystalline lens with age and accommodation. Investigative ophthalmology & visual science.

[21] Atchison DA, Smith G (2004). Possible errors in determining axial length changes during accommodation with the IOLMaster. Optometry and vision science: official publication of the American Academy of Optometry.

[22] Dubbelman M, Van der Heijde GL, Weeber HA, Vrensen GF (2003). Changes in the internal structure of the human crystalline lens with age and accommodation. Vision research.

[23] Le Grand, Y. & El Hage, S. G. Physiological optics. 86–87 (Berlin; New York: Springer-Verlag, 1980).

[24] Gullstrand, A. H I found the mechanism of intracapsular accommodation. *Nobel lecture* (1911).

[25] Fisher RF (1977). The force of contraction of the human ciliary muscle during accommodation. The Journal of physiology.

[26] Sheppard AL, Davies LN (2011). The effect of ageing on *in vivo* human ciliary muscle morphology and contractility. Investigative ophthalmology & visual science.

[27] Wyatt HJ (1993). Application of a simple mechanical model of accommodation to the aging eye. Vision research.

[28] Uhlhorn SR, Manns F, Tahi H, Rol P, Parel JM (1998). Corneal group refractive index measurement using low-coherence interferometry. SPIE.

[29] Atchison DA, Smith G (2005). Chromatic dispersions of the ocular media of human eyes. Journal of the Optical Society of America. A, Optics, image science, and vision.

[30] Eom Y, Kang SY, Song JS, Kim HM (2013). Comparison of the actual amount of axial movement of 3 aspheric intraocular lenses using anterior segment optical coherence tomography. Journal of cataract and refractive surgery.

[31] Modesti M, Pasqualitto G, Appolloni R, Pecorella I, Sourdille P (2011). Preoperative and postoperative size and movements of the lens capsular bag: ultrasound biomicroscopy analysis. Journal of cataract and refractive surgery.

[32] Strenk SA, Strenk LM, Guo S (2006). Magnetic resonance imaging of aging, accommodating, phakic, and pseudophakic ciliary muscle diameters. Journal of cataract and refractive surgery.

[33] Park KA, Yun JH, Kee C (2008). The effect of cataract extraction on the contractility of ciliary muscle. American journal of ophthalmology.

[34] Croft MA (2013). Extralenticular and lenticular aspects of accommodation and presbyopia in human versus monkey eyes. Investigative ophthalmology & visual science.

[35] Koeppl C, Findl O, Kriechbaum K, Drexler W (2005). Comparison of pilocarpine-induced and stimulus-driven accommodation in phakic eyes. Experimental eye research.

[36] Lister LJ, Suheimat M, Verkicharla PK, Mallen EA, Atchison DA (2016). Influence of Gravity on Ocular Lens Position. Investigative ophthalmology & visual science.

[37] Coudrillier B (2012). Biomechanics of the human posterior sclera: age- and glaucoma-related changes measured using inflation testing. Investigative ophthalmology & visual science.

[38] Woodman-Pieterse EC, Read SA, Collins MJ, Alonso-Caneiro D (2015). Regional Changes in Choroidal Thickness Associated With Accommodation. Investigative ophthalmology & visual science.

[39] Friberg TR, Lace JW (1988). A comparison of the elastic properties of human choroid and sclera. Experimental eye research.

[40] Ugarte M, Hussain AA, Marshall J (2006). An experimental study of the elastic properties of the human Bruch’s membrane-choroid complex: relevance to ageing. The British journal of ophthalmology.

[41] Tsorbatzoglou A, Nemeth G, Math J, Berta A (2006). Pseudophakic accommodation and pseudoaccommodation under physiological conditions measured with partial coherence interferometry. Journal of cataract and refractive surgery.

[42] Muftuoglu O, Hosal BM, Karel F, Zilelioglu G (2005). Drug-induced intraocular lens movement and near visual acuity after AcrySof intraocular lens implantation. Journal of cataract and refractive surgery.

[43] Yuan Y, Chen F, Shen M, Lu F, Wang J (2012). Repeated measurements of the anterior segment during accommodation using long scan depth optical coherence tomography. Eye & contact lens.

[44] Shao Y (2013). Simultaneous real-time imaging of the ocular anterior segment including the ciliary muscle during accommodation. Biomedical optics express.

[45] Du C (2012). Anterior segment biometry during accommodation imaged with ultralong scan depth optical coherence tomography. Ophthalmology.

[46] Richdale K (2013). Quantification of age-related and per diopter accommodative changes of the lens and ciliary muscle in the emmetropic human eye. Investigative ophthalmology & visual science.

[47] Strenk SA, Strenk LM, Guo S (2010). Magnetic resonance imaging of the anteroposterior position and thickness of the aging, accommodating, phakic, and pseudophakic ciliary muscle. Journal of cataract and refractive surgery.

[48] Whatham A (2009). Influence of accommodation on off-axis refractive errors in myopic eyes. Journal of vision.

